# Multi-level Risk and Protective Factors for Vaping Onset and Escalation Among Youth: a Focus on LGBTQ + Disparities

**DOI:** 10.1007/s11121-026-01902-6

**Published:** 2026-03-29

**Authors:** Omolayo Anjorin, Anahita Asghari-Kamrani, Lisa Lindley, C. J. Mandell, Rima Nakkash, Kenneth W. Griffin

**Affiliations:** 1 Department of Global and Community Health, George Mason University, Fairfax, VA USA; 2 Department of Community and Global Health, Lehigh University, Bethlehem, PA USA

**Keywords:** LGBTQ + youth, E-cigarette use, Minority stress, Structural stigma, Selective public health interventions

## Abstract

Youth vaping remains a major public health concern, with sexual and gender minority youth experiencing disproportionately higher rates of e-cigarette use compared to their non-LGBTQ + peers. These disparities are not attributable to identity itself, but to social and structural conditions that shape exposure to risk and access to protection. This narrative review synthesizes evidence on shared and LGBTQ + -specific risk and protective factors for vaping initiation and escalation among youth aged 10–24 years. Guided by the Social Ecological Model, findings are organized across individual, interpersonal, organizational/community, and societal levels, and interpreted through two complementary mechanisms: a minority stress–related coping pathway and a socialization and identity pathway. Across levels, discrimination and stigma emerge as cross-cutting risk processes that intensify psychological distress, normalize vaping within social networks, and undermine protective environments. Protective factors, including identity affirmation, supportive relationships, affirming institutions, and inclusive policies, buffer these pathways and promote resilience. This review highlights limitations of one-size-fits-all prevention approaches and underscores the need for selective, mechanism-informed interventions that address both shared developmental risks and the structural drivers of LGBTQ + vaping disparities.

**Supplementary Information:**

The online version contains supplementary material available at 10.1007/s11121-026-01902-6.

## Introduction

E-cigarettes, commonly called vapes, are the most prevalent tobacco product among youth, with an estimated 1.63 million U.S. middle and high school students reporting current e-cigarette nicotine use (CDC, 2021; CDC, 2024). Following World Health Organization (WHO) definitions, “youth” here encompasses ages 10 to 24 to capture key developmental periods for vaping initiation and escalation (World Health Organization: WHO, 2019) (WHO, n.d.) This review focuses exclusively on nicotine-delivering electronic cigarettes, using the terms vaping and e-cigarette use interchangeably. E-cigarette use has been linked to cardiovascular injury, lung damage, impaired immune function, sustained nicotine dependence, and initiation of polysubstance use (Burrow-Sánchez & Ratcliff, 2021; Gaiha et al., 2020; Gilbert et al., 2021; Klein, 2018; Wang & Donaldson, 2023; Wills et al., 2019).

Youth who identify as sexual and gender minorities (Lesbian, Gay, Bisexual, Transgender, Queer, or other minoritized identities, collectively referred to in this review as LGBTQ +) report higher rates of all forms of nicotine use compared to their non-LGBTQ + peers (Acosta-Deprez et al., 2021; Azagba et al., 2014; US Department of Health and Human Services 2024; Raifman et al., 2020; McCabe et al., 2019; Theis et al., 2025). Research has found rates among gay or lesbian (21.5%) and bisexual (18.1%) youth relative higher compared to their heterosexual peers (14.4%) (Azagba et al., 2025). Transgender and gender diverse youth similarly face even more disproportionate risks (Bae et al., 2025; Buchting et al., 2017; Kirby & Yabroff, 2020; McCabe et al., 2024). These disparities mirror broader patterns of substance use inequities, including higher rates of combustible cigarette smoking, alcohol, and cannabis use (Barger et al., 2021; Fahey et al., 2023). Importantly, these disparities are not inherent to sexual orientation or gender identity but reflect social and structural conditions that produce disproportionate exposure to stress and marginalization (Azagba et al., 2014; Office of the Surgeon General, 2024).

### Risk and Protective Factors in Substance Use Among Youth

To understand the persistence of these disparities, this review adopts a risk and protective factors paradigm. Risk factors are defined as conditions that increase the likelihood of e-cigarette use, while protective factors are active assets that foster resilience and buffer against risk, rather than simply representing the absence of risk (Hawkins et al., 1992). Despite the importance of this framework for prevention, most research treats youth as a homogeneous population, often overlooking LGBTQ + -specific mechanisms shaping vaping behavior (Burrow-Sánchez & Ratcliff, 2021; Villanueva-Blasco et al., 2025). By synthesizing LGBTQ + -specific factors alongside those shared with same-age non-LGBTQ + peers, this review informs selective prevention strategies designed specifically for at-risk populations.

### Pathways Underlying LGBTQ + Disparities in Vaping

Risk and protective factors are most effectively understood through the mechanisms by which they influence behavior. This review proposes two complementary etiological pathways through which vaping risk is elevated among LGBTQ + youth: a minority stress-related coping pathway and a socialization and identity pathway.

#### Minority Stress-Related Coping Pathway

The minority Stress Theory defines health disparities among sexual and gender minority populations as the result of chronic, socially produced stressors that accumulate beyond general life stress (Meyer, 2003, 2015). These stressors include distal stressors such as discrimination, bullying, and violence victimization, as well as proximal stressors such as identity concealment, internalized stigma, and expectations of rejection. Persistent exposure to these stressors places sustained demands on coping and emotion regulation, increasing vulnerability to maladaptive coping behaviors, including vaping (Meyer & Frost, 2013).

LGBTQ + youth experience disproportionately higher rates of discrimination, rejection, bullying, and violence victimization compared to their heterosexual and cisgender peers, which act as chronic distal stressors that disrupt emotional self-regulation and increase negative affect (Adamson et al., 2022; Blosnich, 2021). The chronic stigma exposure disrupts emotion regulation and increases negative affect, leading many youths to utilize vaping for stress relief or “self-medication” as a form of maladaptive coping mechanism (Hatzenbuehler, 2009).

#### Socialization and Identity Pathway

The socialization and identity pathway describes how youth are embedded within peer and community contexts where vaping is normalized and symbolically linked to identity expression. While socialization processes influence health behaviors for all youth, this pathway is particularly salient for LGBTQ + youth, who often form tighter and more interconnected social networks in response to exclusion from mainstream spaces (Tse, 2022). Within these networks, peer norms exert stronger influence on behavior. Drawing on social learning, social identity, and diffusion perspectives, this pathway explains how vaping behaviors are observed, adopted, and reinforced through modeling and group affiliation, both in physical and online spaces (Abrams, 2001; Bandura & Walters, 1977; Turner & Reynolds, 2012). In these contexts, vaping may function as a marker of belonging or impression management, while protective behaviors, such as engagement in substance-free spaces, may also diffuse rapidly through shared norms and social reinforcement (Goffman, 1959; Han & Son, 2022).

#### Social Ecological Model as a Theoretical Framework

**Fig. 1:**
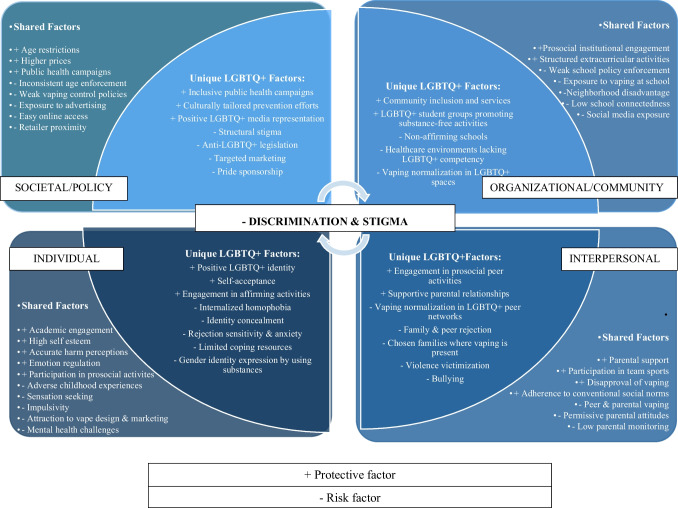
Conceptual model of multi-level influences on LGBTQ + youth vaping risk and protection

This review uses the Social Ecological Model (SEM) to organize vaping-related risk and protective factors across individual, interpersonal, community, and societal levels. The SEM is well suited to the study of health disparities because it conceptualizes behavior as embedded within interconnected social systems (Bronfenbrenner, 1979; McLeroy et al., 1988; Wickman et al., 2025). For instance, individual perceptions of vaping harms are shaped by peer and family norms, which in turn are influenced by broader community and policy environments (Paskett et al., 2016). A conceptual model illustrating these multi-level influences and the intersections of the minority stress and socialization pathways is presented in Fig. 1 (see “Results ” also shown in Supplementary Table 1). Although this review emphasizes selective prevention for LGBTQ + youth, it examines both universal and population-specific processes, recognizing that common developmental risks may be intensified or reshaped by identity-specific social conditions. Distinguishing between shared and LGBTQ + -specific mechanisms clarifies where existing prevention strategies may fall short and where targeted, mechanism-based adaptations are most needed.

## Methods

This narrative review synthesizes evidence on risk and protective factors for e-cigarette use among youth (Green et al., 2006). Electronic databases including PubMed, PsycINFO, and Google Scholar were searched for peer-reviewed literature and public health reports published through July 2025. Search terms included “e-cigarettes,” “vaping,” “nicotine,” “youth,” “sexual and gender minority adolescents,” and “Socio-Ecological determinants.” Electronic databases including PubMed, PsycINFO, and Google Scholar were searched for peer-reviewed literature and public health reports published through July 2025.

## Results

Results are organized by ecological level and interpreted through two overarching pathways: a minority stress–coping pathway, in which stigma-related stress contributes to psychological distress and coping-oriented vaping, and a socialization and identity pathway, in which peer norms, modeling, and belonging shape vaping behaviors. Within each level, shared factors affecting all youth are presented first, followed by LGBTQ + -specific factors that intensify risk.

### Individual Level

#### Shared Risk and Protective Factors

At the individual-level, developmental, cognitive, and psychological characteristics shape vaping behaviors. Sensation seeking, impulsivity, and male gender are consistently associated with higher likelihood of e-cigarette use (Chaffee et al., 2015, 2018; Han & Son, 2022; Lazarus & Folkman, 1984; Meyer, 2003). Early adolescence, particularly ages 14–15, is marked by elevated experimentation, reflecting heightened novelty seeking and risk-taking (Durkin et al., 2021; Usidame et al., 2022). Perceived harm is among the most proximal predictors of vaping. Youth who perceive e-cigarettes as less harmful than combustible cigarettes are significantly more likely to initiate and continue use (Villanueva-Blasco et al., 2025). Among youth aware of e-cigarettes, 34.2% believed they were less harmful than cigarettes, and among those who had tried e-cigarettes, 71.8% held this same belief (Amrock et al., 2015). Product features such as discreet design and appealing flavors further reinforce uptake, particularly among risk-prone youth (Kim et al., 2022). Mental health challenges, including depression, anxiety, and externalizing symptoms, are also significant predictors of vaping initiation and escalation, particularly when co-occurring with prior substance use (Chaffee et al., 2018; Kim et al., 2022). Evidence also suggests associations between e-cigarette use and weight control motives, with youth perceiving vaping as a tool to manage weight (Han & Son, 2022).

Conversely, protective individual-level factors include accurate harm perception, effective emotion regulation, higher self-esteem, academic engagement, positive future orientation, and participation in prosocial activities (Chaffee et al., 2015; Lazarus & Folkman, 1984; Szoko et al., 2021). These factors reduce reliance on maladaptive coping strategies and buffer against escalation.

#### Unique Risk and Protective Factors Among LGBTQ + Youth

LGBTQ + youth experience stress from living in stigmatizing environments, operating through the minority stress pathway (Meyer, 2003). Chronic exposure to discrimination, violent victimization, and exclusion generates proximal stress processes such as identity concealment, internalized stigma, and rejection sensitivity, which are strongly associated with depression, anxiety, and diminished self-worth (Hatzenbuehler, 2009; Meyer, 2003; Newcomb & Mustanski, 2010; Russell & Fish, 2016). These factors increase vulnerability to coping behaviors, including vaping, particularly when safe alternative resources are limited. In terms of socialization, gender identity may further intersect with substance use norms, as some transgender and gender-diverse youth report vaping as aligned with gender expression, with emerging evidence linking medical transition processes to elevated substance use risk (Maglalang et al., 2025).

Protective factors include positive LGBTQ + identity development, self-acceptance, and engagement in affirming activities that promote adaptive coping. Youth who demonstrate resilience in the face of minority stress show lower susceptibility to vaping despite ongoing exposure to stressors (Ceatha et al., 2021; Rosario et al., 2001).

### Interpersonal Level

#### Shared Risk and Protective Factors

Interpersonal relationships are strongly linked to vaping initiation and escalation. Family-level risk factors include parental smoking or vaping, permissive parental attitudes toward e-cigarettes, limited parental monitoring, and exposure to secondhand smoke at home (Kim et al., 2022). Adverse childhood experiences (ACEs) further increase vulnerability to vaping as exposure to parental instability and emotional neglect is associated with higher odds of youth e-cigarette use (Fortier et al., 2022). Peer influence is among the most consistent predictors of vaping initiation, with youth significantly more likely to vape when close friends do so (Burrow-Sánchez & Ratcliff, 2021; Han & Son, 2022; Kim et al., 2022).

In the same vein, protective factors include strong parental monitoring, mother’s education level, and clear disapproval of vaping within peers and families (Aunola & Nurmi, 2005; Han & Son, 2022; Kim et al., 2022). At the peer level, participation in team sports and valuing conventional social norms are associated with reduced e-cigarette (Burrow-Sánchez & Ratcliff, 2021; Han & Son, 2022; Kim et al., 2022; Williams et al., 2021).

#### Unique Risk and Protective Factors Among LGBTQ + Youth

LGBTQ + individuals are often minorities within their biological families, and when family members fail to affirm their identity, the home environment becomes a major source of conflict and psychological distress, representing key interpersonal minority stressors (Matsick et al., 2024; Reczek & Bosley‐Smith, 2021; Van Bergen et al., 2021). One study found that 85% of gay and lesbian individuals described their family’s response to their identity as mixed, containing both supportive and rejecting elements (Reczek, 2016). Even this type of ambivalence led to feelings of rejection and has been associated with poorer mental health outcomes and increased likelihood of using substances like e-cigarettes to cope (Planinac et al., 2024). LGBTQ + youth also experience substantially higher rates of bullying, violence victimization, and peer rejection, which damage self-esteem, harm them and increase reliance on vaping as maladaptive coping (Cooley, 2017; Pettigrew, 1967).

At the same time, LGBTQ + youth frequently form chosen families and close-knit peer networks that provide belonging and support (Boggs et al., 2017). Through the socialization pathway, behaviors modeled within these networks may be adopted to reinforce affiliation and identity. When vaping is normalized within LGBTQ + peer groups, youth may adopt the behavior through observation, reinforcement, and shared norms (Piombo et al., 2025). In these contexts, e-cigarette use may function as a form of impression management**,** allowing youth to signal belonging, visibility, or social competence within LGBTQ + peer spaces, especially if they are excluded from dominant peer spaces (Patterson et al., 2023; Turner, 2001; Turner & Reynolds, 2012).

Conversely, LGBTQ + youth who experience acceptance and affirmation from families are more likely to report positive identity development and lower levels of substance use, including e-cigarettes (Ceatha et al., 2021; Watson et al., 2020). Parental monitoring, open communication, and clear disapproval of vaping have been shown to buffer youth from peer pressure and external stressors (Aunola & Nurmi, 2005; Szoko et al., 2021). Chosen families and affirming peer networks also offer vital protective benefits, especially through the socialization pathway. These serve as safe spaces where LGBTQ + youth can build trust and develop healthy coping strategies (Ceatha et al., 2021; Kim, 2021). Practices such as kinship roles, mentorship, and community-building provide social and emotional stability (Ceatha et al., 2021; Kim, 2021). When health-promoting behaviors are modeled within these networks, they can offset the normalization of vaping and reinforce resilience.

### Organizational and Community Level

#### Shared Risk and Protective Factors

Schools remain the primary organizational setting for youth. School factors, including weak enforcement of vaping bans, and attending under-resourced schools are associated with increased vaping risk (Han & Son, 2022; Ickes et al., 2023; Kaleta et al., 2016; Marion et al., 2021). At the community level, neighborhood economic disadvantage, low social cohesion, and residence in metropolitan areas have also been associated with increased rates of e-cigarette use (Kim et al., 2022; McCabe et al., 2020). Exposure to vaping-related content through digital environments like social media is associated with increased curiosity and initiation among youth (Rutherford et al., 2023).

Protective organizational factors include strong school connectedness and participation in structured extracurricular activities. Institutions that promote inclusive, health-oriented norms and student engagement are associated with lower substance use risk, including vaping (Kim et al., 2022; Szoko et al., 2021).

#### Unique Risk and Protective Factors Among LGBTQ + Youth

Communities often reflect attitudes toward sexual and gender diversity, influencing both exposure to stress and availability of supportive resources. Non-affirming institutional climates, including discriminatory school policies and healthcare settings lacking LGBTQ + competence, increase vaping risk by reinforcing minority stress (Hatzenbuehler, 2009; Ma et al., 2022). In some LGBTQ + community spaces, vaping may be normalized as part of social bonding or identity expression, reinforcing uptake through visibility and normative influence (Linda, 2023; Ghabrial, 2017).

On the other hand, LGBTQ + community organizations that explicitly promote substance-free norms, facilitate engagement in activism, art, mindfulness, and other health-affirming activities offer LGBTQ + youth critical alternatives to vaping as a coping mechanism and foster resilience and improved mental health (Eckstrand & Potter, 2017; Wallace et al., 2024). Other affirming institutions, such as inclusive school environments and supportive healthcare environments, that promote substance-free norms and practice nondiscrimination reduce institutional sources of minority stress. For example, the presence of activities like gay straight alliances (GSA) is associated with improved school connectedness and reduced substance use risk (Seelman et al., 2015).

### Societal and Policy Level

#### Shared Risk and Protective Factors

Weak tobacco control policies, including inconsistent enforcement of minimum age laws and flavored product availability, contribute to increased youth access and initiation (Han & Son, 2022; Kim et al., 2022). Additionally, exposure to e-cigarette advertising, especially through online channels, is strongly associated with increased youth vaping as 13 out of 14 studies in a systematic review found that marketing exposure elevated vaping risk (Han & Son, 2022). Retailer proximity to schools is also a documented risk factor, as is ease of online purchase (Kim et al., 2022).

Protective factors rely on the effective implementation of these policies as studies have shown that robust enforcement of minimum age laws, higher product pricing, and counter-marketing campaigns are associated with reduced youth vaping (Pesko et al., 2018; Reid et al., 2023; Wickman et al., 2025).

#### Unique Risk and Protective Factors Among LGBTQ + Youth

Structural stigma operates through laws, policies, and public discourse that signal exclusion and rejection, amplifying minority stress and vulnerability to coping behaviors such as vaping (Campus et al., 2021; Hatzenbeuhler, 2016). Research found that 79% of LGBTQ + youth said hearing about potential laws to ban conversion therapy made them feel better (Hatzenbeuhler, 2009). Conversely, nearly two-thirds of LGBTQ + youth reported that hearing about potential state or local laws banning discussions of LGBTQ + people in schools worsened their mental health (Hatzenbeuhler, 2009). Such legislative discrimination contributes to an environment where LGBTQ + youth face heightened stress and are more vulnerable to adopting coping behaviors such as vaping. Public health campaigns often fail to recognize these lived realities which in itself is another form of structural stigma. When LGBTQ + identities and risks are overlooked, prevention strategies fall short and existing disparities persist (Hatzenbeuhler, 2016).

Industry marketing practices further amplify risk for LGBTQ + populations (Kickbush et al., 2016). The tobacco industry has a documented history of targeting LGBTQ + individuals, sponsoring Pride events and placing advertisements in LGBTQ + media outlets, including online and social media (Lynn, 2024; Hatzenbeuhler, 2024; Smith et al., 2008). These spaces often serve as critical sources of identity affirmation and peer support for LGBTQ + youth (Ma et al., 2022). Recent research shows that e-cigarette companies have adopted similar tactics, with LGBTQ + youth reporting higher exposure to targeted advertisements (Lewis et al., 2024). While these campaigns are often framed as inclusive outreach, this messaging obscures the exploitative intent of the marketing and reinforces health disparities (Struble et al., 2022; Zenone et al., 2022).

Despite these challenges, inclusive public health campaigns and LGBTQ + -inclusive policies can serve as protective influences by reducing structural stigma and establishing affirming social norms that counter industry narratives (Struble et al., 2022). Protective societal factors also include affirming media representation, and culturally tailored prevention efforts (Ginaldi & Martinis, 2024). These interventions reduce structural stress while disrupting norms that associate vaping with LGBTQ + identity, thereby weakening both the minority stress and socialization pathways.

## Discussion

This review integrates shared and LGBTQ + factors to explain why LGBTQ + youth experience disproportionately high rates of e-cigarette use. While prior reviews have examined risk and protective factors for youth e-cigarette use more broadly, our review makes several distinct contributions. First, it synthesizes factors specific to LGBTQ + youth alongside those that are shared across youth populations. Second, it integrates multiple theoretical perspectives, including minority stress and socialization theories, to clarify the mechanisms underlying LGBTQ + -specific risks. Third, it situates these factors within a socioecological framework to identify potential intervention points across multiple levels of influence. Finally, it centers the implications of these findings for the design of selective prevention interventions tailored to LGBTQ + population (Burrow-Sánchez & Ratcliff, 2021; Villanueva-Blasco et al., 2025; Theis et al., 2025).

Shared risk factors such as peer influence, low perceived harm, and mental health challenges shape vaping behavior across youth. However, the minority stress pathway demonstrates how these risks are intensified for LGBTQ + youth through chronic exposure to stigma, discrimination, and identity-based rejection, demonstrating how stigma-related stress disrupts emotion regulation and increases reliance on vaping as maladaptive coping for LGBTQ + (Hatzenbuehler, 2009; Krueger et al., 2020; Li et al., 2024). Critically, this minority stress pathway, which is operationalized through discrimination, operates across ecological levels.

At the individual-level, findings align with evidence linking sensation seeking, misperceived harm, mental health symptoms, and prior substance use to youth vaping (Chaffee et al., 2015, 2018; Han & Son, 2022; Roditis et al., 2016; Tsai et al., 2018). These mechanisms mirror those observed for alcohol, cannabis, and tobacco use, underscoring that vaping is embedded within broader developmental risk processes (Flay et al., 1999; Hawkins et al., 1992; Meier et al., 2019). For LGBTQ + youth, however, stigma-related stressors such as identity concealment and internalized negativity amplify these risks (McConnell et al., 2018; Meyer, 2003). During adolescence, a critical period for identity creation, vaping may function less as experimentation and more as emotion regulation within the minority stress–coping pathway.

Interpersonal contexts further reveal how shared risks diverge in meaning for LGBTQ + youth. Although family and peer influence are consistent predictors of substance use among youth (Kim et al., 2022), their protective function is conditional for sexual and gender minority youth. Family involvement buffers risk only when accompanied by identity affirmation, whereas conditional acceptance or rejection instead intensifies minority stress (Matsick et al., 2024; Piombo et al., 2025). Similarly, while peer norms influence all youth, LGBTQ + youth excluded from dominant peer groups may seek belonging in alternative networks where vaping is normalized. The socialization pathway highlights how vaping can serve as identity performance or impression management within LGBTQ + spaces, signaling group membership or visibility rather than simple peer pressure (Goffman, 1959).

School connectedness, affirming healthcare, and supportive community organizations are associated with healthier outcomes at the organizational and community level. However, LGBTQ + youth are disproportionately excluded due to non-inclusive policies and provider incompetence (Ickes et al., 2023; Seelman et al., 2015). These institutional gaps simultaneously increase stress exposure and maladaptive coping. In contrast, affirming schools, GSAs, and inclusive healthcare settings weaken both pathways by reducing institutional stress and decoupling identity affirmation from substance use.

At the societal and policy level, structural stigma and targeted marketing emerge as central drivers of disparity (Han & Son, 2022). Weak tobacco regulation and pervasive advertising elevate risk broadly, but LGBTQ + youth are uniquely affected when these forces intersect with discriminatory legislation and exclusionary public discourse (Han & Son, 2022; Hatzenbuehler, 2016). These factors emphasize that disparities are rooted not in individual choice but in systems shaping stress, norms, and access to protection.

### Implications for Prevention Science

Distinguishing shared from population-specific factors explains where existing prevention approaches fall short for LGBTQ + youth. While strategies targeting harm perception and peer resistance address shared developmental risks, they often overlook the fundamental drivers that sustain vaping within marginalized communities. Vaping prevention initiatives must address the minority stress and socialization pathways directly, integrating stress reduction, adaptive coping, affirming relationships, group norm changes, and progressive system-level improvements, and these can be done through selective prevention interventions.

At the individual level, interventions should prioritize emotion regulation and identity affirmation, framing minority stress as a contextual driver. By focusing on this mechanism, programs can help youth recognize and manage instances where vaping is utilized as a primary tool for affect regulation in response to chronic stigma. Similarly, family-based approaches should incorporate explicit care-giver affirmation training, while mentorship within chosen families and affirming peer networks can buffer the effects of biological family rejection.

Organizational strategies at schools and community centers should provide affirming social environments, such as substance-free social venues, that decouple identity expression from vaping or other forms of substance use. Schools can integrate GSA leadership into substance use prevention curricula and anti-discrimination policies; healthcare settings can integrate LGBTQ + cultural competence and vaping prevention into staff training; and community organizations can promote substance-free socialization through LGBTQ + positive activism, art, and collective engagement (Eckstrand & Potter, 2017; Wallace et al., 2024). Finally, societal strategies must address structural stigma through inclusive public health and tobacco policy, counter-industry marketing, supporting substance-free Pride initiatives, and affirming inclusive media representation. By reducing macro-level stressors that place sustained demands on youth coping processes, these policies can effectively weaken the pathways that lead to vaping initiation and escalation.

### Contributions to the Literature

This review advances youth vaping research by integrating minority stress and socialization frameworks to explain how common risk factors take on greater explanatory power for LGBTQ + youth. Unlike prior reviews that document disparities without explicating mechanisms, this synthesis demonstrates how marginalization reshapes both risk exposure and protective capacity (Burrow-Sánchez & Ratcliff, 2021; Villanueva-Blasco, 2025). The review also clarifies protective processes by distinguishing active resilience-promoting factors from the mere absence of risk, highlighting the central role of family acceptance, chosen families, and affirming institutions (Hawkins et al., 1992).

### Limitations and Future Directions

This review has several limitations that should be considered when interpreting its findings. First, as a narrative review, it emphasizes conceptual integration rather than systematic evidence or effect size estimation, limiting conclusions about the strength or consistency of specific associations. Future research can build on this framework to quantify relationships and evaluate intervention effectiveness, including through greater attention to identifying, synthesizing, and testing vaping prevention approaches that are responsive to the needs and contexts of LGBTQ+ youth. Second, the literature is largely cross-sectional, restricting causal inference and understanding of how vaping risk and protective processes unfold over time. Longitudinal research is needed to test the proposed minority stress and socialization pathways across developmental stages.

Third, LGBTQ + youth are often treated as a homogeneous group, obscuring variation across sexual orientation, gender identity, race, and context. Future research should employ disaggregated analyses to identify subgroup-specific mechanisms and intervention needs. Finally, existing research emphasizes individual-level risk, with limited attention to protective processes and structural determinants such as policy environments and targeted marketing. Multilevel studies examining these factors are essential for advancing equity-oriented prevention.

## Conclusion

Vaping among LGBTQ + youth is shaped by the intersection of shared developmental risks and structural conditions rooted in stigma, exclusion, and unequal access to protection. This review demonstrates that disparities are sustained through two interacting mechanisms: minority stress-related coping and socialization tied to identity and belonging. While prevention efforts often focus on individual behavior, these disparities are produced by social and structural forces that amplify stress and normalize maladaptive coping. At the same time, the potential for resilience is substantial. Multilevel protective factors, including affirming relationships, inclusive institutions, substance-free social spaces, and equitable policy environments, represent powerful yet underutilized resources. Reducing vaping disparities requires prevention strategies that move beyond one-size-fits-all approaches and directly address the systems shaping both risk and resilience.

## Supplementary Information

Below is the link to the electronic supplementary material.

ESM 1 (DOCX 15.3 KB)

## Data Availability

Not applicable. No new data were generated or analyzed in this review.

## Declarations

### AI-Assisted Writing Declaration

Artificial intelligence tools were used for language editing and clarity support only. All substantive content, interpretation, and conclusions were generated by the authors, who take full responsibility for the accuracy and integrity of the work.
