# Supplementary material for: Multi-level Risk and Protective Factors for Vaping Onset and Escalation Among Youth: a Focus on LGBTQ + Disparities
Source: Prev Sci. 2026 Mar 29;27(3):478–88. doi: 10.1007/s11121-026-01902-6 (PMC13102936; doi:10.1007/s11121-026-01902-6)
Supplement: Supplementary file 1 — ESM 1 (DOCX 15.3 KB) [file 11121_2026_1902_MOESM1_ESM.docx]

Supplemental Table 1. Multilevel Influences on LGBTQ+ Youth Vaping Risk and Protection

| **Level** | **Shared Factors** | **Unique Factors** |
| --- | --- | --- |
| Societal/Policy | **Protective (+):**  • Age restrictions  • Higher prices  • Public health campaigns  **Risk (−):**  • Inconsistent age enforcement  • Weak vaping control policies  • Exposure to advertising  • Easy online access  • Retailer proximity | **Protective (+):** • Inclusive public health campaigns • Culturally tailored prevention efforts • Positive LGBTQ+ media representation  **Risk (−):** • Structural stigma • Anti-LGBTQ+ legislation • Targeted marketing • Pride sponsorship |
| Organizational/Community | **Protective (+):** • Prosocial institutional engagement • Structured extracurricular activities  **Risk (−):** • Weak school policy enforcement • Exposure to vaping at school • Neighborhood disadvantage • Low school connectedness • Social media exposure | **Protective (+):** • Community inclusion and services • LGBTQ+ student groups promoting substance-free activities  **Risk (−):** • Non-affirming schools • Healthcare environments lacking LGBTQ+ competency • Vaping normalization in LGBTQ+ spaces • Glamorization |
| Interpersonal | **Protective (+):** • Parental support • Participation in team sports • Disapproval of vaping  **Risk (−):** • Peer & parental vaping • Permissive parental attitudes • Low parental monitoring | **Protective (+):** • Engagement in prosocial peer activities • Supportive parental relationships  **Risk (−):** • Vaping normalization in LGBTQ+ peer networks • Family & peer rejection • Chosen families where vaping is present • Violence victimization • Bullying |
| Individual | **Protective (+):** • Academic engagement • High self-esteem • Accurate harm perceptions • Emotion regulation • Participation in prosocial activities  **Risk (−):** • Adverse childhood experiences • Sensation seeking • Impulsivity • Attraction to vape design & marketing • Mental health challenges | **Protective (+):** • Positive LGBTQ+ identity • Self-acceptance • Engagement in affirming activities  **Risk (−):** • Internalized homophobia • Identity concealment • Rejection sensitivity & anxiety • Limited coping resources • Gender identity expression using substances |
